# Wanted—A Revolution in British Nursing

**Published:** 1913-05-10

**Authors:** 


### The Hospital

The Workers' Newspaper of Administrative Medicine and Institutional Life, Administration, National Insurance and Health.

No. 1401, Vol. LIV. SATURDAY, MAY 10, 1913. PRICE ONE PENNY.

### WANTED—A REVOLUTION IN BRITISH NURSING.

It is time to make it impossible for interested parties longer to mislead the public by misrepresentation in nursing matters. The deputation to Mr. Asquith has led to the publication of statements which are notorious and gross exaggerations or demonstrably false. The worst of them could not have been uttered if business methods were to be applied by nurse-training schools, and the training of nurses treated strictly as a matter of business. The truth is that nursing has, like Topsy, "growed" in this country, as nurse registration has "growed" in the United States and sown chaos there. The consequence is that British nursing to-day lacks concentration, and at the best such organisation as exists is due mainly to the efforts of individuals and particular institutions to provide something which originally they required to further the work or objects they had in view. Hence the air at the present time is full of grievance and dissatisfaction, a state of affairs not confined to nursing, it is true, but which in the case of nursing can readily be ended by a peaceful revolution on business lines to the benefit of every individual and every institution concerned.

As we said last week, we owe everything which is entitled to be called training to the nurse-training schools attached to the larger hospitals and a few important Poor-Law infirmaries. Each of these conducts its business in its own way. Registration, and the latter years of training, have been held up to the public, falsely, as a panacea, protection and safeguard on the one hand, and as a grievous form of sweating on the other. The time has arrived when the nurse-training schools should end such evils of misrepresentation and make them impossible by engaging each probationer under a form of contract which sets forth clearly the cost entailed by her training, and the proportion in which that cost will be borne by the school and the probationer, as well as the emoluments to be paid to the latter during her years of training. If the London Hospital, for example, adopted such a contract to-morrow it would be impossible for anyone, however hostile, to sow further prejudice by misrepresentation.

Another storm is brewing, and, we fear, being fermented, against the hospitals who train and employ nurses, through the coming into force of the National Insurance Act. Formerly nurses, it is rather loosely maintained, when working for a hospital, received gratuitously during illness full medical treatment and hospital and convalescent care during the whole period of their inability to continue at work. In fact, though free medical treatment was the rule it was often restricted in quantity, and the invalids, unless very seriously ill, often went to their friends, and did not remain during the whole period of illness under treatment in a hospital or convalescent home. The National Insurance Act compels nurses to insure at a cost of 13s. a year each, and the hospital contributes an equal amount. It is being widely maintained that when a hospital provides an insured nurse with hospital, medical and convalescent treatment it has no right to retain the medical benefit of 8s. 6d. or 9s. nor the sick benefit of 7s. 6d. per week which such a nurse may receive from her approved society. We have no space to-day to enter fully into the questions which underlie these arguments, but it seems to us that the simplest way out of the difficulty would be for each hospital to increase the salary of each nurse by at least 13s. a year. Such a step should remove all sense of hardship, and is far better than trying to meet the case by agreeing that the nurse should receive half her sickness benefit. As to the medical benefit, in those of the larger hospitals we have spoken to this matter is under consideration. It is coupled with the questions of a member of the medical staff being placed on the panel and of the provision of funds to defray the cost of the extra trouble and expense involved.

Grave dissatisfaction is felt, especially in the counties and throughout the country districts, at the shortage of nurses and the unrestricted employment of young women in the smaller hospitals which cannot properly train a single nurse, though they give the untrained women employed the title of probationer and some of them grant certificates of training. It is maintained that the larger nurse-training schools, owing to the enormous developments in methods of treatment, require the majority of the best nurses; each of them trains to make good the wastage in their own staff or to fill up nurses for the paying staff which often brings a large revenue to the hospitals. A very general demand is gathering force to bring pressure to bear upon the nurse-training schools to enlarge the area of their work and greatly to increase the number of probationers admitted to training each year. Failing this a scheme is being evolved to organise independent schools for the training of nurses. These will be organised upon a wide enough basis to provide that every probationer admitted to training shall be guaranteed adequate facilities to enable her to obtain experience in every branch of nursing during the period of her training. A further grievance which is gathering force is that of provincial hospitals which contain enough beds to enable some nurses to be adequately trained, but the certificates of which do not carry the weight of one granted by a large training-school.

We have merely indicated a few of many aspects of the nursing question which press for solution. It is felt by some of the most trusted and hardest worked of nursing authorities in this country that an absolute necessity exists for a careful consideration and a thorough reorganisation of the education and training of nurses in this country at the present time. We should like to see one of the bigger schools take the lead by instituting new contracts of employment for probationers and forming a small committee of the most active and knowledgeable of the heads of British nurse-training schools to inquire into the whole of these matters and to prepare a report. National Insurance must result in serious modifications to our hospital system, and it is of the first importance that this fact should be recognised by all who are chiefly responsible for the training of nurses. It is time that the best brains of those engaged in this work should be behind an organised movement to prepare the way to bring about a modern up-to-date and ample scheme of nurse-training to embrace all grades of nurses throught the country.

